# Contrasting Microbiome Dynamics of Putative Denitrifying Bacteria in Two Octocoral Species Exposed to Dissolved Organic Carbon (DOC) and Warming

**DOI:** 10.1128/AEM.01886-21

**Published:** 2022-01-25

**Authors:** Nan Xiang, Christiane Hassenrück, Claudia Pogoreutz, Nils Rädecker, Susana Marcela Simancas-Giraldo, Christian R. Voolstra, Christian Wild, Astrid Gärdes

**Affiliations:** a Alfred Wegener Institute, Helmholtz Centre for Polar and Marine Research, Bremerhaven, Germany; b Marine Ecology Department, Faculty of Biology and Chemistry, University of Bremen, Bremen, Germany; c Leibniz Centre for Tropical Marine Research, Bremen, Germany; d MARUM–Center for Marine Environmental Sciences, University of Bremen, Bremen, Germany; e Department of Biology, University of Konstanz, Konstanz, Germany; f Laboratory for Biological Geochemistry, School of Architecture, Civil and Environmental Engineering, École Polytechnique Fédérale de Lausanne (EPFL), Lausanne, Switzerland; g Hochschule Bremerhaven, Bremerhaven, Germany; Shanghai Jiao Tong University

**Keywords:** nitrogen limitation, denitrification, microbial plasticity, Symbiodiniaceae-bacteria interactions, phase shifts

## Abstract

Mutualistic nutrient cycling in the coral-algae symbiosis depends on limited nitrogen (N) availability for algal symbionts. Denitrifying prokaryotes capable of reducing nitrate or nitrite to dinitrogen could thus support coral holobiont functioning by limiting N availability. Octocorals show some of the highest denitrification rates among reef organisms; however, little is known about the community structures of associated denitrifiers and their response to environmental fluctuations. Combining 16S rRNA gene amplicon sequencing with *nirS in-silico* PCR and quantitative PCR, we found differences in bacterial community dynamics between two octocorals exposed to excess dissolved organic carbon (DOC) and concomitant warming. Although bacterial communities of the gorgonian *Pinnigorgia flava* remained largely unaffected by DOC and warming, the soft coral *Xenia umbellata* exhibited a pronounced shift toward *Alphaproteobacteria* dominance under excess DOC. Likewise, the relative abundance of denitrifiers was not altered in *P. flava* but decreased by 1 order of magnitude in *X. umbellata* under excess DOC, likely due to decreased proportions of *Ruegeria* spp. Given that holobiont C:N ratios remained stable in *P. flava* but showed a pronounced increase with excess DOC in *X. umbellata*, our results suggest that microbial community dynamics may reflect the nutritional status of the holobiont. Hence, denitrifier abundance may be directly linked to N availability. This suggests a passive regulation of N cycling microbes based on N availability, which could help stabilize nutrient limitation in the coral-algal symbiosis and thereby support holobiont functioning in a changing environment.

**IMPORTANCE** Octocorals are important members of reef-associated benthic communities that can rapidly replace scleractinian corals as the dominant ecosystem engineers on degraded reefs. Considering the substantial change in the (a)biotic environment that is commonly driving reef degradation, maintaining a dynamic and metabolically diverse microbial community might contribute to octocoral acclimatization. Nitrogen (N) cycling microbes, in particular denitrifying prokaryotes, may support holobiont functioning by limiting internal N availability, but little is known about the identity and (a)biotic drivers of octocoral-associated denitrifiers. Here, we show contrasting dynamics of bacterial communities associated with two common octocoral species, the soft coral *Xenia umbellata* and the gorgonian *Pinnigorgia flava* after a 6-week exposure to excess dissolved organic carbon under concomitant warming conditions. The specific responses of denitrifier communities of the two octocoral species aligned with the nutritional status of holobiont members. This suggests a passive regulation based on N availability in the coral holobiont.

## INTRODUCTION

Coral reefs are hot spots of marine biodiversity and primary productivity in oligotrophic tropical oceans (1). Corals, the ecosystem engineers of these reefs, are key to supporting these ecosystems (2). The symbiosis with intracellular dinoflagellate algae of the family Symbiodiniaceae is central to this ecological success as it enables corals access to heterotrophic as well as autotrophic nutrient sources to supporting growth and productivity (3, 4). In particular, the translocation of organic carbon (C) in the form of photosynthates by symbiotic algae is a major energy source for the coral host, which provides inorganic nutrients and carbon dioxide from its catabolism to supporting algal photosynthesis (5). However, the efficient symbiotic trade of C in the coral-algae symbiosis depends heavily on limited nitrogen (N) availability for the algae. Constant N limitation of algal symbionts is required to limit their populations and ensure the accumulation of excess photosynthates available for translocation (5, 6). Given the pronounced environmental fluctuations and seasonality in coral reefs, the functioning of symbiosis thus depends on active and/or passive regulation of nutrient availability for algal symbionts, summarized in (7). Importantly, the nutrient availability in the symbiosis does not depend on interactions of the host and its symbiotic algae alone (8, 9). Corals also associate with a diverse prokaryotic microbiome with varying degrees of taxonomic flexibility depending on host species and environmental conditions (10). Many members of the prokaryotic microbiome are or may be actively involved in the provision and recycling of limiting nutrients such as N, phosphorus (P), thereby altering nutrient availability for the holobiont (11–14). As such, the ecological success of corals likely depends on an intricate functional interplay of all its microbial associates. This diverse multispecies assemblage termed the coral holobiont extends the metabolic properties of its members and may help their rapid adaptation to changing environmental conditions (15).

For millions of years, the functional interplay between holobiont members has formed the basis of the ecological success of corals and the reefs they support (16–18). Yet in recent decades, anthropogenic activities have led to widespread coral mortality and reef degradation (19, 20). Global and local stressors such as ocean warming and labile DOC loading are known to disrupt holobiont functioning, resulting in coral bleaching (i.e., the collapse of the coral-algal symbiosis) or coral diseases (21–23). The breakdown of this symbiosis is not exclusively restricted to interactions between the coral host and their algal symbionts but involves other members of the holobiont as well (e.g., prokaryotes) (10, 24, 25). Given that the stability of the coral-algal symbiosis is dependent on N limitation of the algal symbionts, microbial N cycling may stabilize or destabilize holobiont functioning depending on the environmental conditions (7, 14, 26).

In particular, denitrifiers, that is, prokaryotes that encompass the reduction of nitrate or nitrite to dinitrogen gas (27), might help alleviate excess N stress of holobionts (7). Denitrifiers appear to be widely associated with many reef organisms and their activity was recently confirmed in corals from the Red Sea (28). Specifically, it has been observed that the activity of microbial denitrification in coral holobionts increased with environmental N levels (29). As such, changes in community structure, abundance, and activity of denitrifiers might directly affect coral holobiont functioning by altering N availability for other holobiont members. Yet, it remains unexplored how environmental changes, such as ocean warming and excess DOC, influence coral-associated denitrifying bacterial communities.

**FIG 1:**
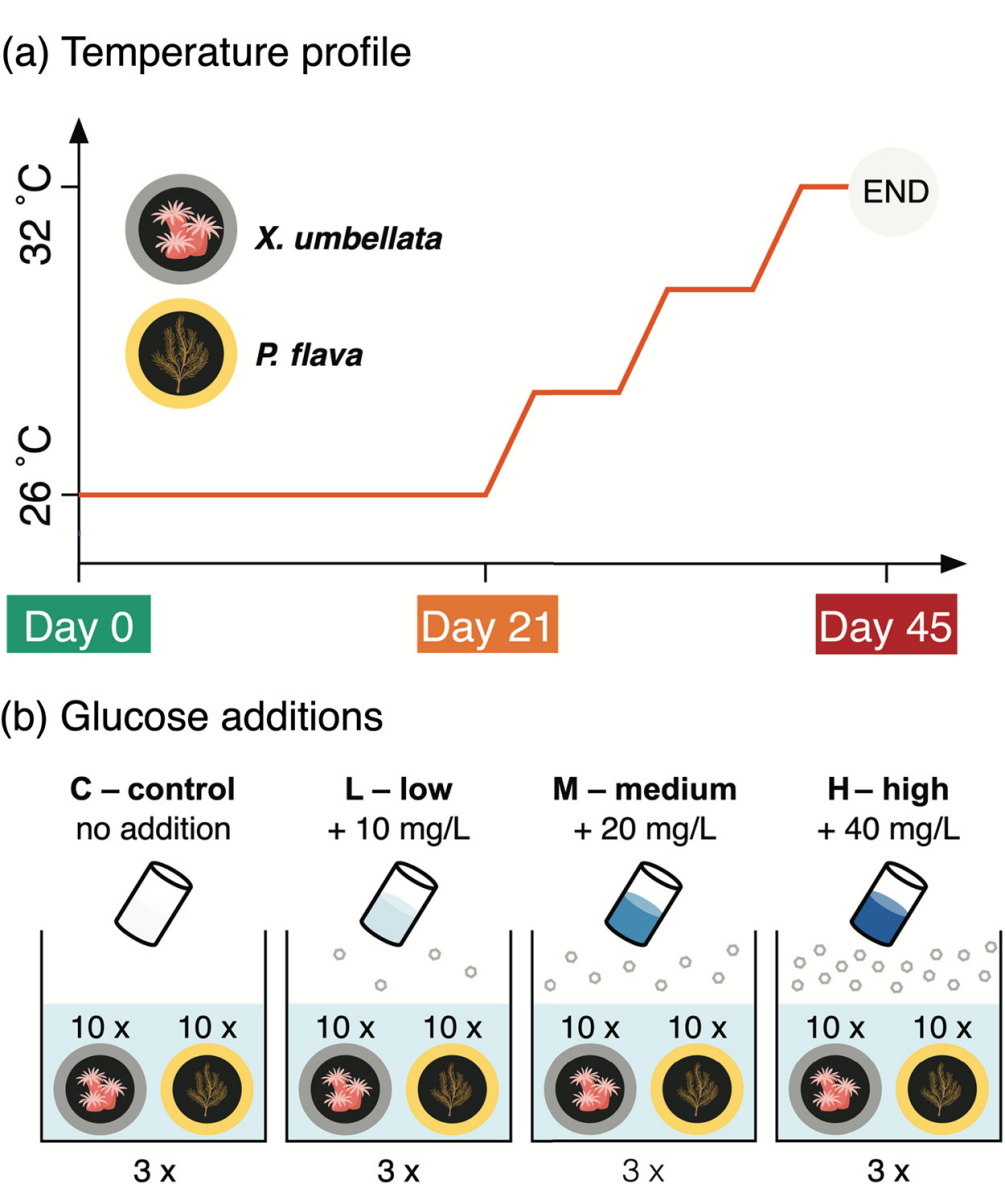
Design of the conducted manipulative aquarium experiment. (a) Step-wise increases in temperature started from day 21 to day 45 with an increase in 2°C every 8 days. (b) Dissolved organic carbon (i.e., glucose) additions were applied daily throughout the experiment.

To date, most studies have focused on the bacterial community dynamics of scleractinian corals given their importance as reef ecosystem engineers (25, 30). In contrast, even though octocorals constitute highly abundant members of benthic communities of coral reefs, their bacterial community dynamics under environmental stress remain largely unexplored (31). Octocorals were recently shown to exhibit some of the highest denitrification rates among Red Sea reef organisms and substrates (32), indicating a potential importance of this microbial functional trait to octocoral health and holobiont functioning. However, the (a)biotic drivers of denitrifier abundance and community composition in octocoral holobionts are unknown. Here, we set out to explore the effects of excess DOC and concomitant warming on bacterial community structure with a focus on diversity and abundance of putative denitrifiers associated with the pulsating soft coral *Xenia umbellata* (Lamarck, 1816) and the gorgonian *Pinnigorgia flava* (Nutting, 1910). The experiment consisted of two consecutive phases. In phase 1, excess DOC enrichment was performed over the course of 21 days at varying concentrations via administration of daily glucose dosing. In phase 2, the continued DOC enrichment was combined with stepwise warming for 24 days (Fig. 1). By doing so, we aimed to (i) provide a comprehensive overview of the bacterial symbionts associated with two common and taxonomically distinct octocoral species and their response to excess DOC and ocean warming; and (ii) provide a comparative assessment on the abundance, diversity, and community dynamics of associated putative denitrifiers in these octocoral holobionts.

## RESULTS

### Overview of 16S rRNA gene sequencing data.

Over the duration of 45 days of the aquarium experiment with two octocoral species, using four levels of DOC concentrations and two different temperatures, a total of 78 coral fragments were collected for 16S rRNA gene amplicon sequencing on the Illumina MiSeq platform. Of these, 76 samples passed quality control, resulting in 8,145 amplicon sequence variants (ASVs) based on 1,150,262 sequences. Of total ASVs, 5,703 ASVs were present in both coral species. 1,847 ASVs were exclusively associated with the soft coral *X. umbellata* and 595 ASVs with the gorgonian *P. flava* only. Under undisturbed condition, the total number of bacterial ASVs associated with *X. umbellata* (6,344 ASVs at day 0) was higher than in *P. flava* (4,260 ASVs at day 0). However, over the course of our experiment, *X. umbellata* showed a decline in total ASV diversity regardless of DOC treatments (1,618 and 1,058 ASVs for day 21 and 45, respectively), as well as significantly reduced Inverse Simpson Index (two-way ANOVA, time-effect: F_2, 24_ = 14.97, *P < *0.001, DOC-effect: F_4, 24_ = 5.76, *P = *0.002). Specifically, compared to day 0, the Inverse Simpson Index experienced an 80% reduction at day 21 (Tukey's honestly significant difference [HSD], *P = *0.003) and an 84% reduction at day 45 (Tukey's HSD, *P < *0.001) (Fig. S1 and Fig. S2 in the supplemental material). In contrast, ASV numbers and Inverse Simpson Index remained stable in the *P. flava* bacterial microbiome (Two-way ANOVA, time-effect: F_2, 26_ = 1.57, *P = *0.227, DOC-effect: F_4, 26_ = 0.20, *P = *0.936; Fig. S1 and Fig. S2) throughout the experiment.

### Distinct bacterial communities and bacterial community responses of *X. umbellata and P. flava*.

**FIG 2:**
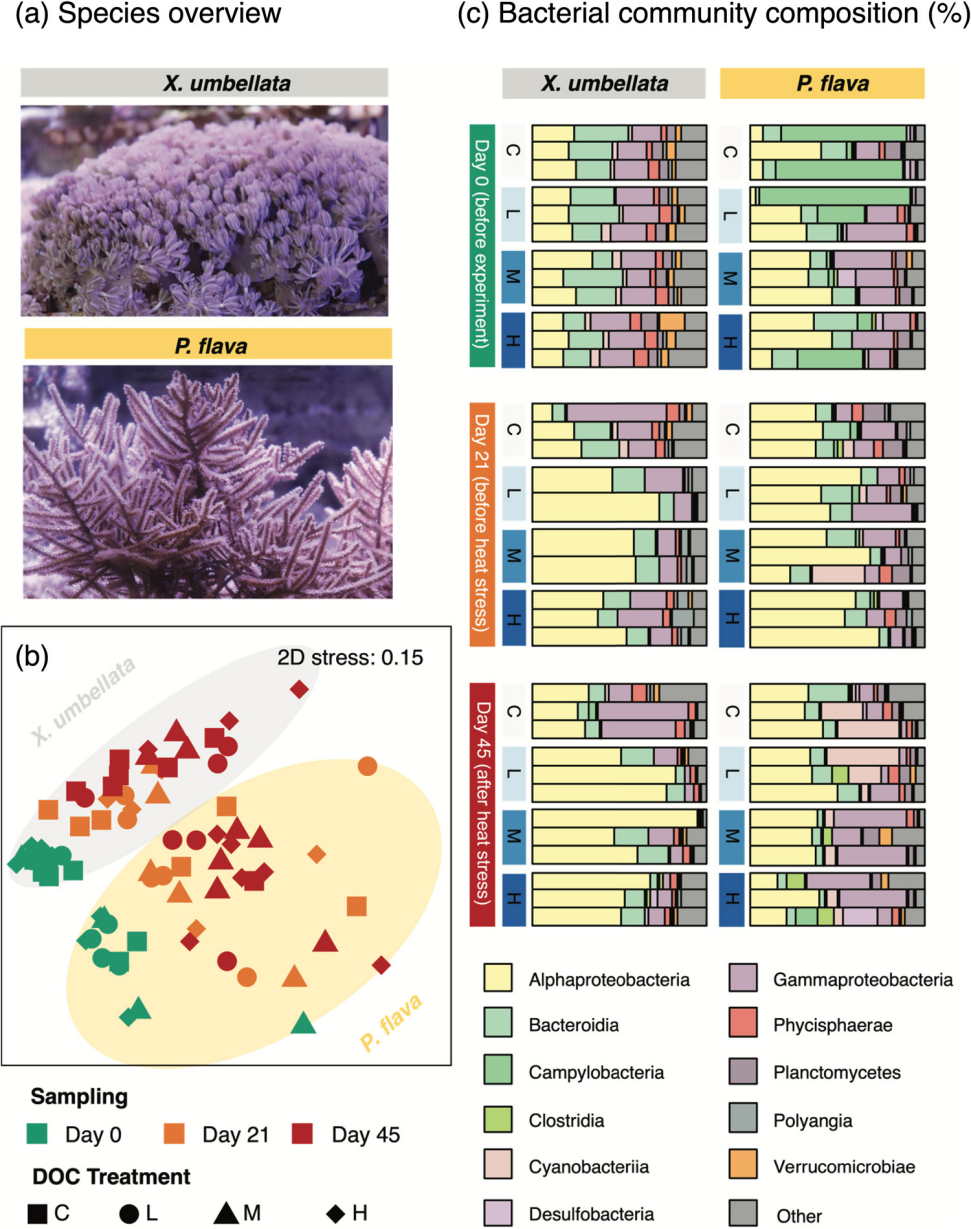
Bacterial community compositions of corals *X. umbellata* and *P. flava* over the course of the experiment. (a) Representative photographs of corals. (b) Non-metric multidimensional scaling (NMDS) plot based on Bray-Curtis dissimilarity matrix of bacterial community compositions associated with coral samples at day 0, day 21, and day 45. (c) Stacked bar plots of bacterial community compositions of corals across different time points and DOC treatments. Stacked bar plots display the 10 most abundant bacterial classes (>1%, determined for each coral species separately).

At day 0, that is, just prior to the start of the experiment, both octocoral species were associated with distinct dominant bacterial taxa (analysis of similarities [ANOSIM], *R* = 0.68, *P = *0.001; Fig. 2b, c). In *X. umbellata*-associated bacterial communities, *Alphaproteobacteria* (25–30% proportion; ca. 70% of sequences affiliated to *Rhodobacteraceae*), *Bacteroidia* (20–25% proportion; ca. 75% of sequences affiliated to *Kordia*) and *Gammaproteobacteria* (15–25% proportion; ca. 70% of sequences affiliated to *Alteromonadaceae*) were identified as dominant classes. In contrast, *P. flava* was dominated by *Alphaproteobacteria* (20–40% proportion; ca. 65% of sequences affiliated to *Rhodobacteraceae*), *Gammaproteobacteria* (10–40% proportion; ca. 16% of sequences affiliated to *Vibrionaceae*) and *Campylobacteria* (15–80% proportion; ca. 70% of sequences affiliated to *Arcobacteraceae*) (Fig. 2c).

*X. umbellata* bacterial communities significantly varied across time points (PERMANOVA, F_2, 22_ = 5.07, *P = *0.001), and were significantly affected by excess DOC (PERMANOVA, F_3, 22_ = 1.92, *P = *0.003) (Fig. 2c). At day 21, bacterial community structure of *X. umbellata* at control did not markedly change from day 0 (ANOSIM, *R* = 0.52, *P = *0.132; Fig. 2c). Yet, bacterial communities under excess DOC separated well from the control (ANOSIM, *R* = 1.00, *P = *0.132; Fig. 2c) by showing a 2-fold increase in the proportion of *Alphaproteobacteria*, primarily ASVs affiliated to *Rhodobacteraceae* (63%–75% proportion of *Alphaproteobacteria*) and *Hyphomonadaceae* (25–28%). Until day 45, following continuous excess DOC paired with a temperature increase to 32°C, *X. umbellata* bacterial communities were dominated by *Alphaproteobacteria* (mainly *Rhodobacteraceae*; 75–80% of all sequences in this class), a higher proportion than in corals with excess DOC at day 21 (ANOSIM, *R* = 1.00, *P = *0.132; Fig. 2c)*. P. flava-*associated bacterial communities differed significantly over time (PERMANOVA, F_2, 24_ = 2.60, *P = *0.001). This was first and foremost driven by the 8-fold increased proportion of ASV5, which was affiliated with *Paraspirulinaceae*, in corals without excess DOC. In contrast, the bacterial community structure of *P. flava* did not significantly respond to excess DOC throughout the experiment (PERMANOVA, F_3, 24_ = 0.99, *P = *0.500; Fig. 2c).

### Pronounced effects of excess DOC on composition and abundance of putative denitrifiers associated with *X. umbellata*.

**FIG 3:**
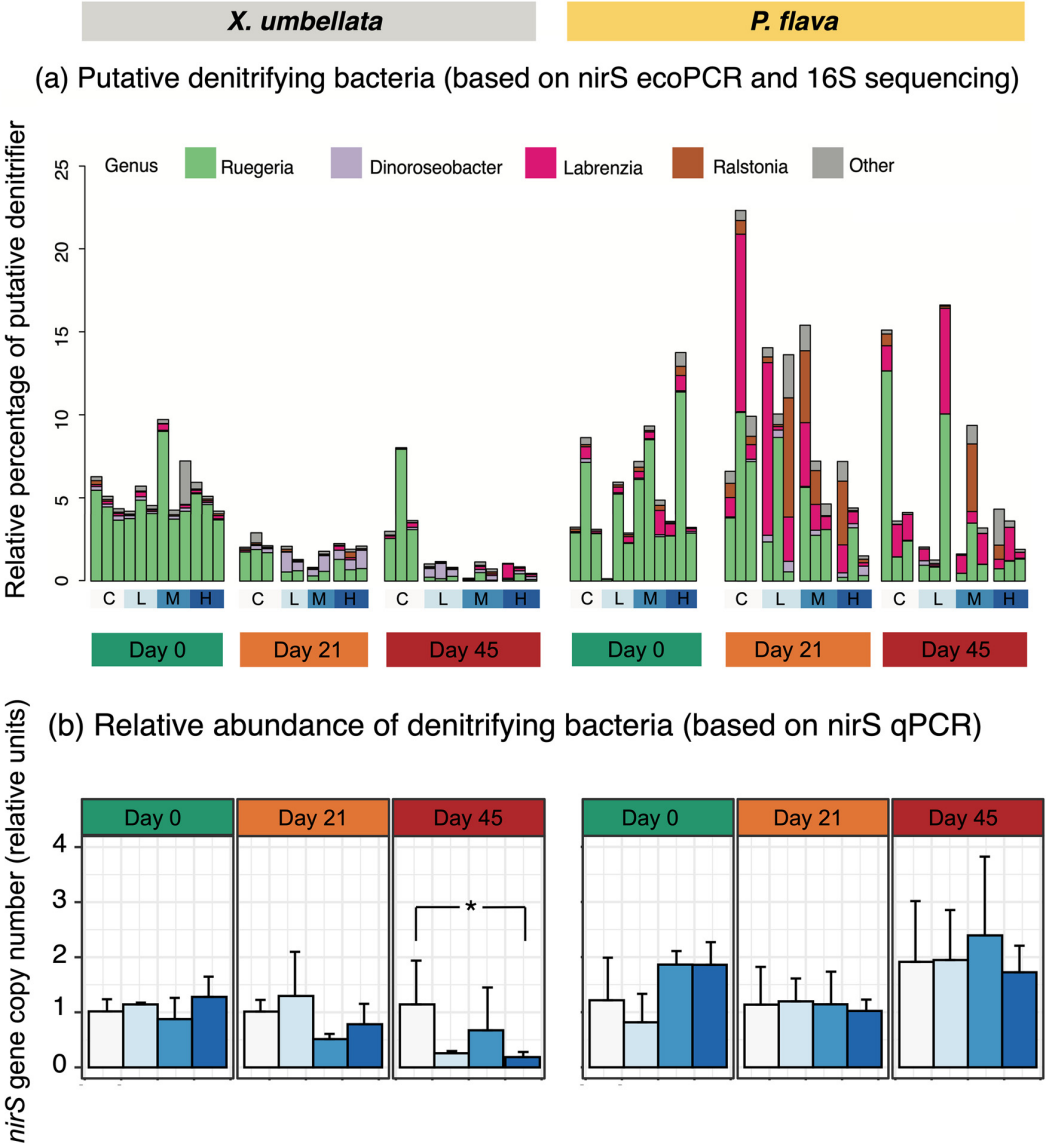
Community compositions and relative abundances of putative denitrifiers in corals *X. umbellata* and *P. flava* over the course of the experiment. (a) Relative proportions of denitrifier genera of corals *X. umbellata* and *P. flava* inferred by *nirS in-silico* PCR in relation to the total bacterial community from 16S rRNA gene sequencing. (b) Relative fold changes in copy numbers of *nirS* gene referenced to 16S rRNA gene and in relation to the day 0 control samples (*n* = 3) of corals *X. umbellata* and *P. flava*. Values are means ± SD, and the asterisk indicates statistically significant differences (*P < 0.05).

The *nirS in-silico* PCR and 16S rRNA gene sequencing retrieved a total of 97 ASVs distributed over 14 putative denitrifying genera from 76 octocoral samples. Putative denitrifying taxa were represented by 12 genera and 75 ASVs and accounted for approximately 10% of the *X. umbellata* bacterial community. The putative denitrifying community in *P. flava* varied more across samples than in *X. umbellata*, with denitrifiers being represented by 14 genera from 79 ASVs, accounting for up to 23% of the overall bacterial community. *Ruegeria* (ca. 90% of denitrifier-affiliated sequences, across 23 ASVs dominated by 60–65% ASV8 and 26–35% ASV11) showed its dominance in both octocoral denitrifying communities at day 0. *Labrenzia* (including ASV43, ASV126, and ASV179) was the second most dominant genus and occupied a higher proportion in *P. flava* (Fig. 3a).

The denitrifying community of *X. umbellata* revealed a ca. 80% reduction in sequence proportion related to *Ruegeria* spp. and a ca. 30% increase in the proportion of sequences affiliated with *Dinoroseobacter* spp. after 21 days of excess DOC (Fig. 3a). However, this shift did not affect the cumulative relative abundance of putative denitrifiers, as estimated by *nirS* gene relative abundance (ANOVA, F_3, 24_ = 2.280, *P = *0.105; Fig. 3b). Denitrifying community structure in *P. flava* showed no significant response to DOC, as well as a non-significant change in the *nirS* relative abundance based on qPCR (ANOVA, F_3, 24_ = 0.961, *P = *0.427; Fig. 3b).

**FIG 4:**
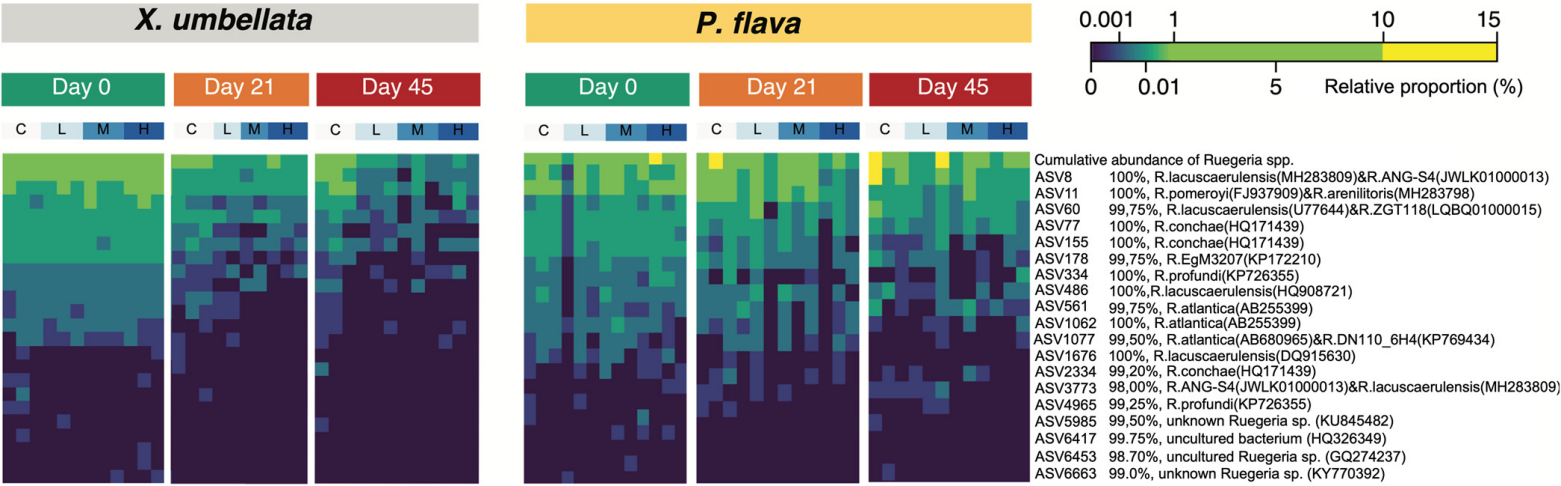
Relative proportions of putative denitrifying *Ruegeria* spp. ASVs in total bacterial communities associated with corals *X. umbellata* and *P. flava* across different time points and DOC treatments.

The denitrifiers of *X. umbellata* revealed significant changes in community structure across treatments at day 45. This was primarily due to the reduced proportion of *Ruegeria* spp. under excess DOC and concomitant warming (Fig. 3a, Fig. 4). This shift in the community was further reflected in a significant decrease in *nirS* relative abundance in corals under excess DOC (ANOVA, F_6, 24_ = 2.45, *P = *0.05; Fig. 3b), especially when comparing the high DOC treatment (at a concentration of 40 mg L^−1^) to the control (Tukey's HSD, *P = *0.03). Conversely, denitrifiers in *P. flava* showed no significant change in community structure or cumulative relative abundance (*nirS* qPCR, ANOVA, F_6, 24_ = 0.93, *P = *0.49; Fig. 3b) in the presence of excess DOC and warming.

### Physiological changes in *X. umbellata* subjected to excess DOC and warming.

**FIG 5:**
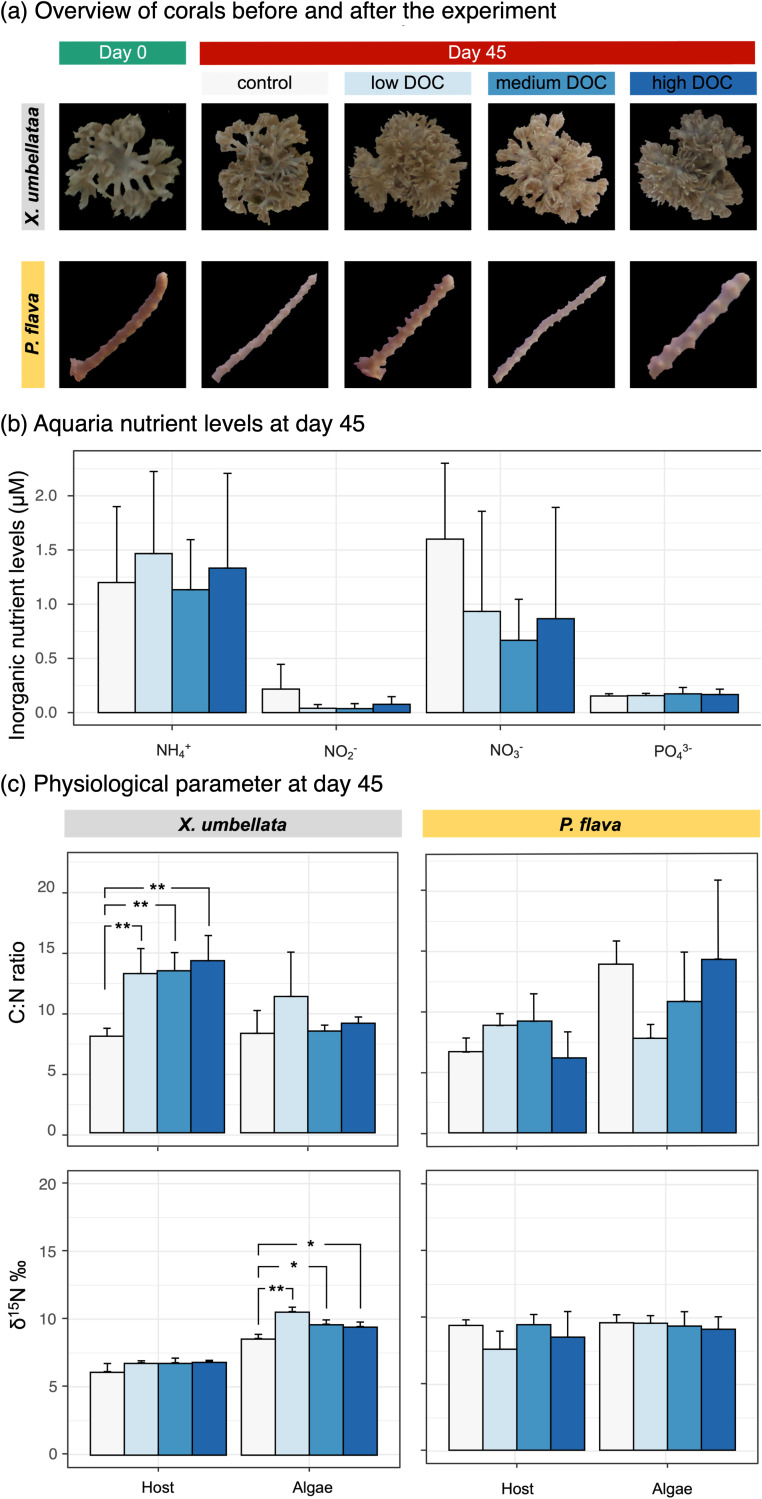
Physiological changes in corals *X. umbellata* and *P. flava* at the end of the experiment. (a) Phenotypes of corals *X. umbellata* and *P. flava* before and after the experiment. (b) Seawater inorganic nutrient levels in all aquaria at the end of the experiment. (c) Elemental (carbon and nitrogen) changes in both coral holobionts. Values are means ± SD, and the asterisk indicates statistically significant differences (*P < 0.05, **P < 0.01).

No change in phenotype was observed for the two octocoral species with excess DOC for 21 days. Even at the end of the experiment after combined excess DOC and warming conditions, *X. umbellata* still maintained a healthy appearance across excess DOC groups (Fig. 5a). In contrast, moderate bleaching was observed in *P. flava* under higher DOC concentrations (i.e., 20 mg L^−1^, 40 mg L^−1^; Fig. 5a). At day 45, *X. umbellata* with excess DOC showed 30% higher C:N ratios in host tissues compared to their control counterparts (ANOVA, F_3, 8_ = 12.59, *P = *0.002; Fig. 5c), while no significant difference was observed in C:N ratios of Symbiodiniaceae (ANOVA, F_3, 8_ = 1.002, *P = *0.44; Fig. 5c). This was accompanied by a moderate albeit not significant increase in host δ^15^N signatures (ANOVA, F_3, 8_ = 2.7, *P = *0.116), as well as a 25% increase in δ^15^N signatures of Symbiodiniaceae under excess DOC (ANOVA, F_3, 8_ = 19.44, *P < *0.001; Fig. 5c). There was no statistically significant difference across treatments in C:N ratios of *P. flava* host (ANOVA, F_3, 8_ = 2.435, *P = *0.14) and Symbiodiniaceae (ANOVA, F_3, 8_ = 2.508, *P = *0.133), nor in δ^15^N‰ signatures of host (ANOVA, F_3, 8_ = 1.396, *P = *0.313) and Symbiodiniaceae (ANOVA, F_3, 8_ = 0.234, *P = *0.87) (Fig. 5c).

At the end of the experiment, seawater nutrient levels did not significantly differ among treatments with regard to NH_4_^+^ (ANOVA, F_3, 8_ = 0.069, *P = *0.975) and PO_4_^3-^ (ANOVA: F_3, 8_ = 10.08, *P = *0.969) levels (Fig. 5b). Nevertheless, NO_2_- (0.22 ± 0.19 μM) and NO_3_- (1.60 ± 0.57 μM) concentrations from the control were 2- to 4-fold higher (NO_2_-, ANOVA, F_3, 8_ = 1.502, *P = *0.286; NO_3_-, ANOVA, F_3, 8_ = 0.741, *P = *0.557; Fig. 5b) than those of aquaria with excess DOC (NO_2_-: 0.05 ± 0.04 μM; NO_3_-: 0.84 ± 0.66 μM).

## DISCUSSION

N cycling microbes, including denitrifiers, are considered key players in the coral holobiont, as they might help in maintaining a N-limited state critical for the functioning of the coral-algae symbiosis (7, 33, 34). In the current study, we provide an assessment of the overall bacterial and denitrifying community structures associated with two octocoral species as well as their abundances and dynamics under excess DOC and concomitant warming in a 6-week aquarium experiment. We found pronounced octocoral species-specific responses to excess DOC in bacterial community structure in general, and in denitrifier community composition and abundance, which aligned with patterns in C:N ratios of host tissues. Taken together, our results suggest a link between octocoral-associated denitrifiers and nutritional status (i.e., N availability of the holobiont), as well as a passive regulation of denitrifier communities as discussed below.

### Distinct bacterial community structures between octocoral species.

Bacterial community structure and diversity were distinct between two octocoral species *X. umbellata* and *P. flava* at the beginning of the experiment, even though these corals had been maintained in the same aquaria for years. At the bacterial class level, *P. flava* was initially dominated by *Alpha*- and *Gammaproteobacteria* as well as *Campylobacteria*; *X. umbellata* was dominated by *Alphaproteobebacteria*, *Bacteroidia*, and *Gammaproteobacteria*. Our results are in line with previous findings that octocoral-associated bacterial communities are species-specific (35–38). This also concurs with the abundantly reported host-specific microbial assemblages for scleractinian corals, as well as a structural similarity to scleractinian coral bacterial communities at the class level (15, 25, 30, 39, 40). The gorgonian *P. flava* showed a lower bacterial diversity, which is consistent with previous reports (36, 37). Due to the broad leaf-like morphology and comparatively small polyps, algal symbionts may maximize photosynthetic activity to sustain a high autotrophic capacity in gorgonians (41). The invariable C:N ratio in the *P. flava* holobiont in our study suggests a relatively poor heterotrophic capacity, lending support to previous observations by Baker et al. (41). The low heterotrophic capacity of *P. flava* may restrict bacterial diversity (42). Limited niche space for bacterial associates due to morphological and physiological features of *P. flava* may be responsible for the observed low bacterial diversity. Being an octocoral, *P. flava* does not build a porous calcium carbonate skeleton that may offer niche space for a number of specialized endolithic microbes, as known from scleractinian corals (43, 44), nor does it secrete a thick surface mucus layer like *Xenia* sp. (45) that may provide favorable habitat for prokaryotic growth, including that of anaerobes (46, 47).

### Species-specific responses of octocoral-associated bacterial communities to excess DOC.

Little information on drivers of octocoral microbial dynamics is available. Here we showed that octocorals exhibit species-specific responses to excess DOC and concomitant warming with regard to their bacterial communities. Over time, excess DOC led to conspicuous changes in the bacterial community structure of *X*. *umbellata*, as characterized by a marked continuous increase in *Rhodobacteraceae*, making up to 80% of the overall bacterial community at the end of the experiment. We cannot disentangle the direct and indirect effects of DOC on associated microbiomes. While based on qPCR data of 16S rRNA gene, we found an overall increase of bacterial abundance in *X. umbellata* under excess DOC. In contrast, no such change in response to excess DOC was observed in the bacterial community structure or abundance of *P. flava*. The absence of change in bacterial community structure in *P. flava* aligns with prior reports that gorgonian corals host a group of extremely robust bacterial communities over large geographic scales (48), seasonal variations (37) and even in the presence of environmental stressors such as increased temperatures and UV radiation (35). These observations suggest a differential “flexibility” or plasticity in bacterial community responses, as recently reported for a number of scleractinian corals (49).

This apparent interspecific plasticity of octocoral microbiomes may be explained by the initial differences in bacterial diversity and community structure between these two octocoral species. In general, coral bacterial microbiomes with greater diversity likely show a higher degree of functional redundancy and may exhibit more flexible associations in response to environmental change, which align with the ecological resilience of the holobiont (10, 25, 50–52). In contrast, the lower diversity of the bacterial community associated with *P. flava* might suggest bacteria are highly selected and likely inhabit host-constructed niches, as previously reported (53). These may be dominated by a few specialized species of high abundance (49) and vary less under environmental perturbation (54).

Observations of such a species-specificity may pertain to functional traits of the animal host and/or algal symbionts, such as heterotrophic capacity, nutritional status, or ecological resilience, including but not limited to heat tolerance as well as (a)biotic environmental drivers (25, 54–56). Differences in trophic strategies between these two octocoral species in particular may be of potential importance for their contrasting responses of the overall bacterial community under excess DOC. *Xenia* sp. is thought to be entirely autotrophic (57). However, Fabricius and colleague argued that *Xenia* sp. may exhibit a mixotrophic lifestyle, that is, relying on autotrophic and heterotrophic food sources (58). Our findings of increased C:N ratios in *X. umbellata* under excess DOC lend support to the latter. Indeed, the capacity to feed heterotrophically may allow *X. umbellata* to benefit from the surrounding DOC, thereby also creating a favorable environment for heterotrophic bacterial propagation, which may subsequently alter the overall bacterial community structure.

*X. umbellata* is known for its distinctive pulsation behavior, which may contribute to its ecological resilience by mixing of the surface boundary layer (59, 60). Of note, excess DOC remarkably enhanced their pulsation rates (61) and net primary productions (62), but whether this increase in pulsation may have an effect on the microbial plasticity of *X. umbellata* remains to be determined. Some endosymbiotic Symbiodiniaceae are known to feed heterotrophically (63), suggesting that excess DOC in *X. umbellata* may also promote the heterotrophic growth of Symbiodiniaceae. Given Symbiodiniaceae-associated bacteria often form a major component of the coral microbiome (64, 65), changes in Symbiodiniaceae physiology or even community composition likely contribute to microbial community dynamics observed in the present study.

Distinct patterns of microbial plasticity between two octocoral species appeared to be primarily driven by the increase of *Rhodobacteraceae* (ASV1: Roseobacter clade CHAB-I-5; ASV9 and ASV13: unclassified Rhodobacteraceae) exclusively in *X. umbellata*. This observation contrasts the findings of (49) reporting on the structural inflexibility in the bacterial microbiome of the scleractinian coral *Pocillopora verrucosa* under excess DOC. The pronounced increase in sequences proportions affiliated to multiple *Rhodobacteraceae* ASVs however align well with genomic evidence and ecological observations (67). *Rhodobacteraceae* are known for their considerable versatility with regard to C utilization, which allows them to thrive and rapidly proliferate in high-DOC environments (67). Due to the intragenomic heterogeneity, 16S rRNA gene sequencing using hypervariable region V3-V4 could overestimate the prokaryotic diversity of our investigated octocoral species (77). However, this overestimated diversity caused negligible effects on our main conclusion. As we compared samples across the same species under different conditions, the relative error remained the same and the differences are still a reflection of differences in treatments.

### A passive regulation of denitrifiers in octocoral holobiont functioning.

Coral-associated diazotrophs (N-fixing prokaryotes) have previously been suggested to play a central role in supporting holobiont fitness and functioning when the surrounding environmental N availability is low (7, 26). In contrast, denitrifiers have been proposed to be important in maintaining the coral-algae symbiosis in a N-limited state (7), yet our understanding of whether their abundance in the coral holobiont is of physiological or ecological relevance remains poor. On average, the proportion of putative denitrifiers in the microbiome was higher in *P. flava* than in *X. umbellata*. This difference between host species may be attributed to their distinct trophic strategies. Microbial community compositions in coral holobionts are highly selective due to different host functional or life history traits pertaining to e.g., development, physiology, and metabolism (17, 40, 68). Due to a relatively low capacity to obtain (in)organic N sources from the surrounding environment, *P. flava* is likely to rely more on symbiotic N cycling microbes to acquire or remove N to fulfill its metabolic requirements (13, 28, 69).

In the presence of N, bioavailable C sources including glucose usually favor denitrification and hence the growth of denitrifiers (70). However, excess glucose caused no stimulating effects on the relative abundance of denitrifiers in our study. This apparent contradiction might be explained by the following two considerations: first, denitrifier populations might be regulated by N availability; consequently, denitrifier abundance may not necessarily increase in the presence of high DOC loads in a N-limited environment. Second, if denitrifiers were not limited by environmental N availability, different denitrifying taxa may exhibit differential preferences for C sources. For instance, some *Rhodobacteraceae* taxa dominate microbial glucose uptake in coastal North Sea waters (71), while others may be suppressed by allochthonous glucose input, but follow fluctuations in population dynamics of primary producers (72).

Importantly, a reduction in the cumulative relative abundance of denitrifiers (as reflected in *nirS* relative gene copies quantified by qPCR) during DOC enrichment was exclusively observed in *X. umbellata*. In contrast to *P. flava*, this soft coral species showed an increase in C:N ratios in the animal host under excess DOC at the end of the experiment. As increased C:N ratios imply a relative decrease of N availability, associated changes in the nutritional status of the host may directly impact its interaction with associated denitrifiers. The notion of reduced N availability for holobiont members is further corroborated by the increase in δ^15^N signatures, potentially indicating a reduced uptake of inorganic N from the seawater and an increased retention of N within the holobiont.

A passive regulation of N cyclers could thereby directly support overall holobiont functioning under fluctuating environmental conditions. In periods of low N availability, reduced denitrifier abundance (and therefore overall denitrification activity) might reduce the competition for N source in the coral holobiont and favor N uptake by the algae to support their growth. Likewise, rapid growth of denitrifiers during periods of excess N availability could increase the competition for N source between holobiont members, thereby alleviating excess N stress, and ultimately stabilizing the coral-algal symbiosis.

### Can denitrifiers provide new insights into Symbiodiniaceae-bacteria interactions?

By inference, all four dominant denitrifiers identified in our study appear to form close associations with Symbiodiniaceae instead of coral hosts. The predominant putative denitrifier *Ruegeria* spp., formerly named *Silicibacter* spp., are known as dinoflagellate-associated bacteria. They are attracted to and capable of catabolizing degrading DMSP produced by the dinoflagellate host (73). *Labrenzia* spp. were previously reported to be core microbiome members of Symbiodiniaceae in the coral holobiont (64, 65). Given their ability to produce DMSP (an osmolyte and powerful scavenger of reactive oxygen species), the consistent association of *Labrenzia* spp. potentially assist in reducing oxidative stress of Symbiodiniaceae (65). Likewise, *Dinoroseobacter* spp. were previously shown to supply their dinoflagellate host with essential nutrients, specifically the essential vitamins B_1_ and B_12_, to support their growth in particular under the nutrient-limitation state (74). *Ralstonia* spp. were identified as intracellular, i.e., occurring within the endodermal coral host cells in close proximity to the Symbiodiniaceae, and were proposed to be implicated in the functioning of the coral-algae symbiosis (53).

Notably, we observed differential responses of different putative denitrifying taxa to experimental treatments. Specifically, an increasing proportion of *Dinoroseobacter* spp. concurs with a decreasing proportion of *Ruegeria* spp. in *X. umbellata* with excess DOC. At this point, the causes and consequences of altered denitrifier community structure and abundance for octocoral holobiont functioning remain to be determined. Functional studies will be required to link these microbiome dynamics with related denitrifying activities and nutritional states of holobionts to disentangle the potential role of denitrifiers in octocoral holobiont fitness and functioning. Further localization of denitrifiers in the intact symbiosis could help us better understand the interactions between octocoral-associated N cycling microbes with other members of the holobiont: the animal host, Symbiodiniaceae, and other microbes in a changing environment.

### Conclusion.

Phase shifts on coral reef ecosystems have been linked to several environmental stressors. Among those, the detrimental effects of excess DOC (typically associated with sewage and reduced water quality) on scleractinian corals have received considerable attentions in the past (21, 22, 75). Changes in the activity, abundance and community structure of N cycling microbes have been discussed as critical components to corals’ response to environmental DOC loading. While an imbalance in N cycling significantly affected the structure and functions of bacterial microbiomes associated with scleractinian corals, very little information is available for octocorals, which are globally abundant particularly on reefs undergoing phase shifts (66, 76). Here, we provide insights into the dynamics of octocoral-associated bacterial communities with an emphasis on putative denitrifying communities under excess DOC and warming. The dynamics of denitrifiers aligned with the nutritional status of the octocoral host, which implies their critical role in regulating internal nutrient availability of the holobiont in a changing environment. To obtain a better understanding on the interactions between octocoral holobiont members, future studies should expand to a comparative taxonomic framework of octocoral host species and link their functional and life history traits to their microbial communities, in particular potentially critical functional groups such as N cyclers. Integrated holistic approaches combining “-omics” approaches and cultivation-dependent methods may aid such a challenging endeavor.

## MATERIALS AND METHODS

### Coral preparation and maintenance.

The soft coral *X. umbellata* and the gorgonian *P. flava* were cultivated (temperature: 26 ± 0.5°C; pH 7.8 ± 0.2; salinity: 35 ± 3‰) for more than 2 years at the Marine Ecology Department of the University of Bremen. Coral species were identified by barcoding gene (*COI*, *mutS* and 28S rRNA gene) amplification and sanger sequencing. Small *X. umbellata* fragments (1-2 cm in side length) were cut from 5 mother colonies (5 × 7×12 cm) and fixed on cubical-shaped calcium carbonate coral holders (1 × 1 cm) with rubber bands. After fragments recovered from fragmentation for 7 days and attached to the holders, rubber bands were removed. Simultaneously, branches of 3–4 cm in height were cut from 4 mother colonies (18 × 1×24 cm) of *P. flava*, and subsequently attached to coral holders using aquarium moss coral fix glue. Thereafter, 120 fragments of *X. umbellata* and 120 fragments of *P. flava* were distributed among 12 experimental aquaria tanks (water volume 50 L) and acclimated for 10 days prior to the experiment. Each aquarium was equipped with a thermostat (3613 aquarium heater, 75 W 220–240 V), a pump (EHEIM Compact On 300 pump) and a protein skimmer that were all purchased from EHEIM GmbH and Co. KG in Germany to maintain stable aquarium conditions. Additionally, LED lights (Royal Blue-matrix module and Ultra Blue White 1:1-matrix module, WALTRAt day time LED light, Germany) were used to simulate day - night rhythm of 12 h-12 h at the intensity of 120.8 ± 10.2 μmol quanta m^−2^ s^−1^. No additional feeding was provided and 10% of the artificial seawater was exchanged daily to maintain stable water parameters. To verify stable environmental conditions, salinity (C: 35.39 ± 0.05; L: 35.38 ± 0.08; M: 35.50 ± 0.08; H: 35.11 ± 0.09) and pH (C: 8.15 ± 0.01; L: 8.05 ± 0.02; M: 8.05 ± 0.02; H: 8.03 ± 0.02) were monitored throughout the experiment (Fig. S4 in the supplemental material).

### Experimental design, sampling and DNA extraction.

In the first phase of experiment, temperature was kept constant at 26°C for 21 days (Fig. 1). Daily additions of glucose to the aquaria based on a stock solution (d-Glucose, 40 mg ml^−1^) were used to simulate four different glucose enrichment levels: control (no addition), low (equivalent to 10 mg L^−1^), medium (equivalent to 20 mg L^−1^) and high (equivalent to 40 mg L^−1^). Daily measurements of total organic carbon (TOC) using a TOC-L analyzer (Shimadzu, Japan) were used to approximate glucose levels in the aquaria and the daily dosing of glucose was adjusted accordingly to achieve the desired enrichment levels. For the second phase, temperature was increased gradually in all aquaria, ultimately adding 2°C every 8 days to a final temperature of 32°C. The range of 26–32°C represents a latitudinal gradient of mean maximum temperature from north to south across the Red Sea, where *X. umbellata* is widely abundant (78). The temperature maximum of 32°C is also close to the thermal physiological limit of this species (79). During this gradual ramping phase, all DOC treatments were continued as described above (Fig. 1). The experiment was terminated at day 45.

Corals for molecular analysis were collected at day 0 (before DOC additions, as baseline data), after the first phase of the experiment (day 21, DOC treatments at 26°C), and after the second phase (day 45, DOC treatments at 32°C). For this, fresh coral samples were collected and immediately frozen in liquid nitrogen and stored at −80°C until further processing. Additionally, samples for physiological measurements and aquarium seawater inorganic nutrients were collected at day 45 as outlined below. Frozen coral samples were ground into powder over liquid nitrogen using mortar and pestle. Genomic DNA was extracted according to the instruction of Quick-DNA Universal Kit Quick Protocol for Solid Tissue (ZYMO RESEARCH, USA). Afterwards, DNA was quantified by spectrophotometry at 260 nm and 280 nm (Tecan Infinite 200 PRO, Austria) and quality-checked by 1% (wt/vol) agarose gel electrophoresis (Biometra Horizon 58, Germany).

### Illumina MiSeq 16S rRNA gene sequencing and sequence analysis.

The 16S rRNA gene amplicon sequencing was conducted at LGC genomics (Berlin, Germany). The hypervariable regions V3-V4 of the bacterial 16S rRNA gene were amplified and sequenced using the primer pair S-d-Bact-0341-b-S-17 (5′-CCTACGGGNGGCWGCAG-3′) and S-d-Bact-0785-a-A-21 (5′-GACTACHVGGGTATCTAATCC-3′) (80). The PCRs included 1–10 ng of DNA extract (total volume 1 μl), 15 pmol of each forward primer and reverse primer in 20 μl volume of 1 x MyTaq buffer containing 1.5 units MyTaq DNA polymerase (Bioline GmbH, Germany) and 2 μl of BioStabII PCR Enhancer (Sigma-Aldrich Co., Germany). For each sample, the forward and reverse primers had the same 10-nt barcode sequence for multiplexing. PCRs were carried out for 35 cycles using the following parameters: 1 min 96°C predenaturation; 96°C denaturation for 15s, 55°C annealing for 30s, 70°C extension for 90s, hold at 8°C. The Illumina library was pooled, and size selected by preparative gel electrophoresis, and the sequencing was conducted on the Illumina MiSeq platform in a 2 × 300 bp paired-end run using V3 Chemistry.

After demultiplexing and removal of primer sequences from the raw paired-end reads by LGC genomics, further sequence processing was performed according to the DADA2 (1.14.1) pipeline for the generation of exact amplicon sequence variants (ASVs) (81). Specifically, sequences were filtered and quality trimmed to 225 bp (forward) and 235 bp (reverse) at a maximum expected error rate of 5. Trimmed sequences were pooled and used for error learning and denoising. In total, 77 samples were pooled with 3,858,215 reads in 1,264,025 unique sequences. Denoised sequences were merged, followed by chimeras’ removal according to default parameters. A total of 26,902 chimeras were identified out of 40,839 ASVs, singletons generated during the merging step were removed. ASVs between 400 to 430 bp were retained and taxonomically classified by “assignTaxonomy” based on the SILVA database release 138 (82). ASVs that matched chloroplast and mitochondrial sequences were removed prior to further analysis.

An *in silico* PCR for the nitrite reductase *nirS* primer pair, nirS-1F (5′-CCTAYTGGCCGCCRCART-3′) and nirS-qR (5′-TCCMAGCCRCCRTCRTGCAG-3′) (83) was used to characterize the putative denitrifier community. The program ecoPCR (84) from OBI tools 1.01.22 was launched against the Ensembl Bacteria release 42 with a maximum three mismatches and a zero mismatch zone of 2 bp at the 3′ end of each primer, retaining fragments between 50 bp and 500 bp. Resultant sequences from ecoPCR with the fragment size between 224 and 227 bp were blasted against the GenBank Nucleotide database (NCBI nucleotide BLAST, date accessed 2020/04/05), and sequences that were not identified as originating from denitrifying *nirS* were removed. The genus affiliation of the remaining *nirS* fragments was used as potential denitrifying taxa to recover denitrifier communities based on our 16S rRNA gene sequencing results. In addition, ASV sequences affiliated to the predominant genus were aligned to SILVA 138, and the accession numbers of nearest relatives from SILVA Incremental Aligner (SINA) output were used to obtain a higher resolved taxonomic path.

### Quantification real-time PCR (qPCR) of denitrifying *nirS* gene.

We assessed the denitrification potential in the coral holobiont via the relative quantification of *nirS* gene, which catalyzes the conversion of nitrite to nitric oxide in the denitrification cascade (85) and has been previously used to determine denitrifier abundance and diversity (27, 83, 86). The *nirS* gene was amplified using the same primer pair *nirS*-1F, qR (83) previously used for *in silico* PCR as outlined above, and validated with Sanger sequencing (StarSEQ, Mainz, Germany). The relative quantification of *nirS* gene abundance was done by qPCR using the CFX96^TM^ Touch Real-Time PCR Detection System (BIO-RAD, USA) by SensiFAST™ SYBR No-ROX Kit (Bioline, USA). C_T_ values of *nirS* gene amplicons (as a proxy of denitrifier abundance) were referenced against C_T_ values of 16S rRNA gene amplicons (as a reference for total bacterial abundance) using the primer-pair Bact-16S_784F: 5′-AGGATTAGATACCCTGGTA-3′ and Bact-16S_1061R: 5′-CRRCACGAGCTGACGAC-3′ (87), according to the delta-delta Ct method (2^-ΔΔCt^) (88). 10-20 ng of DNA extract was used for 16S rRNA gene and *nirS* qPCRs. Final cycling conditions consisted of a hot-start activation at 95°C for 2 min, followed by 40 cycles of denaturation at 95°C for 10 s, annealing at 65°C (*nirS* gene in *X. umbellata*) or 60°C (*nirS* gene in *P. flava* and 16S rRNA gene for both species) for 20 s, and extension at 72°C for 30 s. Final extension was carried out at 72°C for 10 s followed by a melting curve from 65 to 95°C with increase of 0.5°C steps every 5 s. The qPCR efficiency was validated by calibration curves of genomic DNA from E. coli (ATCC 25922) targeting 16S rRNA gene and DSM 428 *Alcaligenes eutropus H16* targeting *nirS* gene separately.

### Seawater inorganic nutrient and coral elemental analysis.

Nutrient samples were collected in triplicates at the end of the experiment (i.e., day 45). 50 ml of aquaria seawater was collected through 0.45 μm filters in 50 ml sterilized centrifuge tubes, and immediately frozen at −20°C until further analysis. Nutrient levels were measured spectrophotometrically using the Infinite 200 PRO (Tecan Infinite 200 PRO, Austria) according to reference 89. Coral samples for elemental and isotope analysis were collected at day 45. Fresh coral fragments were immediately frozen in liquid nitrogen and stored at −80°C until further processing. Corals were thawed at room temperature and homogenized for 30 s at 3,500 rpm with an Ultra Turrax (IKA, Germany). The resulting homogenized coral slurry was separated into coral tissue and algal symbiont fractions by centrifugation at 3,000 × *g* for 5 min (Eppendorf, Germany). The host fraction, that is, the resulting supernatant, was carefully removed by pipetting without disturbing the algal symbiont pellet. Algal symbiont pellets were resuspended in 0.22 μm filtered seawater (FSW) in sterilize 2 ml Eppendorf tubes, and host and algal samples were dried at 40°C for 1 week. After the dried matter was pulverized using clean mini pestles; 1.0 mg of coral tissue or algal symbiont sample was used for measuring total nitrogen content (TN). Further, 1.0 mg of coral tissue or algal symbiont sample mixed with 200 μl 1 mol L^−1^ HCl was used for analyzing total organic carbon content (TCorg). TN and TCorg were analyzed in an elemental analyzer (Euro EA, Germany), Isotopic δ^15^N and δ^13^C signatures were measured using an Isotope Ratio MS (Thermo Fisher, USA), following reference 90.

### Statistical analysis.

All statistical analyses were performed in R studio 3.6.1, specifically *vegan* for multivariate statistics (91) and *ggplot2* for visualization (92). Alpha diversity indices were calculated based on repeated (*n* = 100) random subsampling of ASVs to the minimum library size at sequencing depth 3,000. Data normality was determined by the Shapiro–Wilk test, and statistical differences between different time points and DOC treatments for each species was tested using two-way analysis of variance (ANOVA) with Tukey’s HSD as a *post hoc* comparison. Beta dispersion of samples was conducted for each coral species using function “betadisper”. Beta diversity was evaluated between species, time points and DOC treatments by non-metric multidimensional scaling (NMDS) plot based on Bray-Curtis dissimilarities of relative ASV proportions. Analysis of similarities (ANOSIM) was used to illustrate the dissimilarity of bacterial community structures between two coral species at day 0. To illustrate the significant difference in bacterial variations at ASV level from different time points and DOC treatments, permutational multivariate analysis of variance (PERMANOVA, ‘adonis’ function, 999 permutations) based on Bray-Curtis dissimilarity was applied along with ANOSIM as *post hoc* comparisons. An ANOSIM R value close to 1 suggests a strong separation between groups, while values close to 0 indicates an overlap between groups (93). After log transformation to meet data normality, homogeneity of variance and independence, the qPCR data was analyzed by two-way ANOVA and Tukey’s HSD. Seawater inorganic nutrient levels in aquaria and elemental changes in animal host and algal symbionts respectively were analyzed by one-way ANOVA and Tukey’s HSD.

### Data availability.

The Sanger sequencing data derived from *COI*, *mutS*, and 28S rRNA gene PCR amplicons for octocoral identification have been deposited in the European Nucleotide Archive (ENA) under the project accession number PRJEB43824. Primer-clipped DNA sequences generated by Miseq 16S rRNA amplicon sequencing were deposited on NCBI under BioProject accession number PRJNA718022.
