# Supplementary material for: Contrasting Microbiome Dynamics of Putative Denitrifying Bacteria in Two Octocoral Species Exposed to Dissolved Organic Carbon (DOC) and Warming
Source: Appl Environ Microbiol. 2022 Jan 25;88(2):e01886-21. doi: 10.1128/AEM.01886-21 (PMC8788706; doi:10.1128/AEM.01886-21)
Supplement: Supplemental file 1 — Fig. S1 to S4. Download AEM.01886-21-s0001.pdf, PDF file, 2.1 MB [file aem.01886-21-s0001.pdf]

Supporting Figure legends

Fig. S1 Rarefaction curves of bacterial communities in corals *X. umbellata* and *P. flava*. (a) ASV numbers with sequencing depth of 200,000 in *X. umbellata* and ASV numbers with sequencing depth of 80,000 in *P. flava*. (b) ASV numbers at the minimum library size with sequencing depth of 3,000 in corals *X. umbellata* and *P. flava*.

Fig. S2 Alpha diversity of bacterial communities at the minimum library size with sequencing depth of 3,000 in corals *X. umbellata* and *P. flava*. The asterisks indicate statistically significant differences compared to day 0 for each species separately (*$p < 0.05$; **$p < 0.01$; ***$p < 0.001$). (a) Chao1 index. (b) Inverse Simpson Index. (c) ACE index. (d) Shannon diversity index.

Fig. S3 Multivariate dispersion of bacterial communities in corals *X. umbellata* and *P. flava*. The asterisks indicate statistically significant differences between groups (*$p < 0.05$; **$p < 0.01$; ***$p < 0.001$).

Fig. S4 The fluctuations in salinity (daily measurement) throughout the experiment. Values are shown by means ± SE for different treatments: (a) control; (b) low DOC (10 mg L^{-1}); (c) medium DOC (20 mg L^{-1}); (d) high DOC (40 mg L^{-1}).

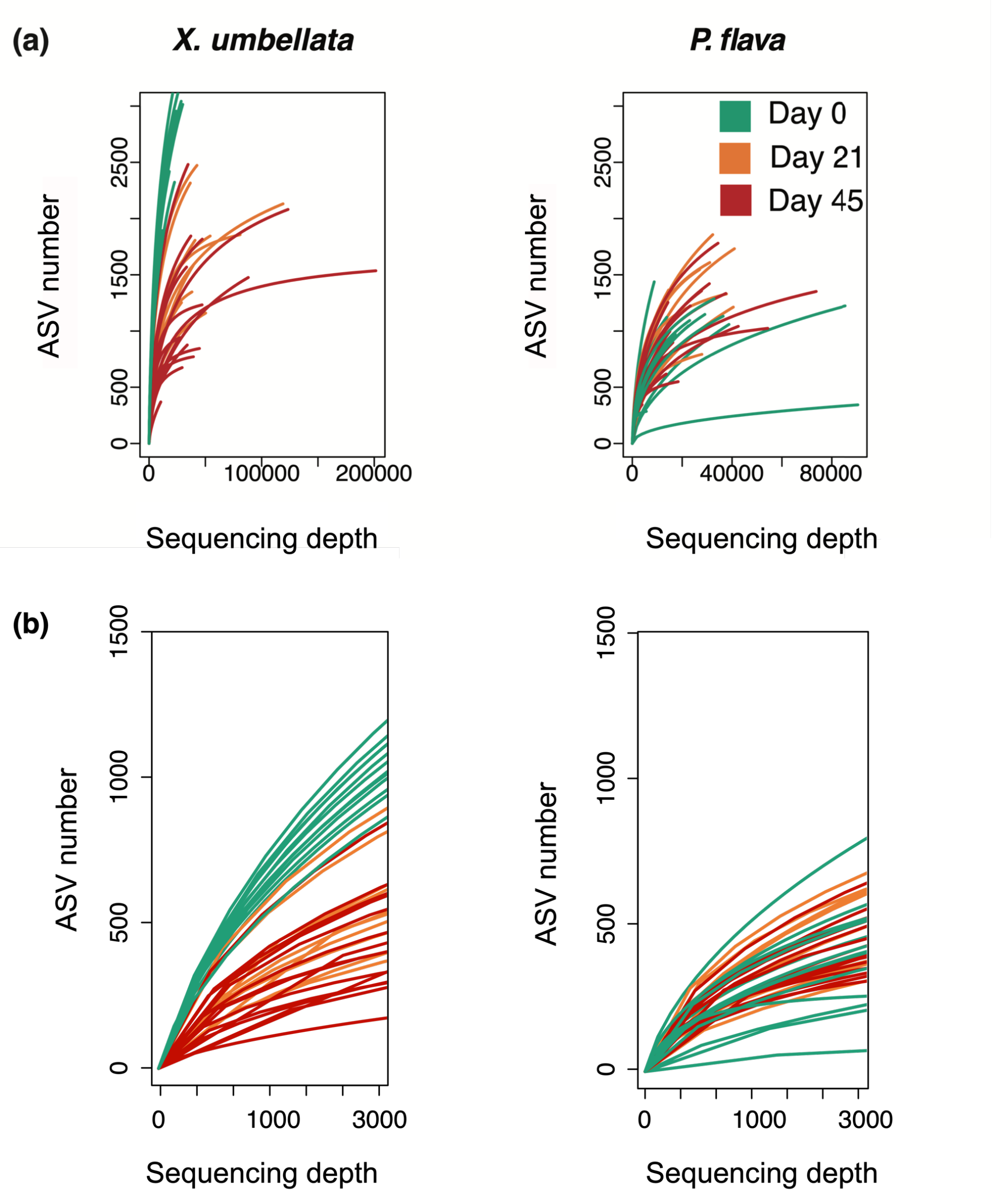

Fig. S1 Rarefaction curves of bacterial communities in corals *X. umbellata* and *P. flava*. (a) ASV numbers in *X. umbellata* with sequencing depth of 200,000 and in *P. flava* with sequencing depth of 80,000. (b) ASV numbers in corals *X. umbellata* and *P. flava* with sequencing depth of 3,000.

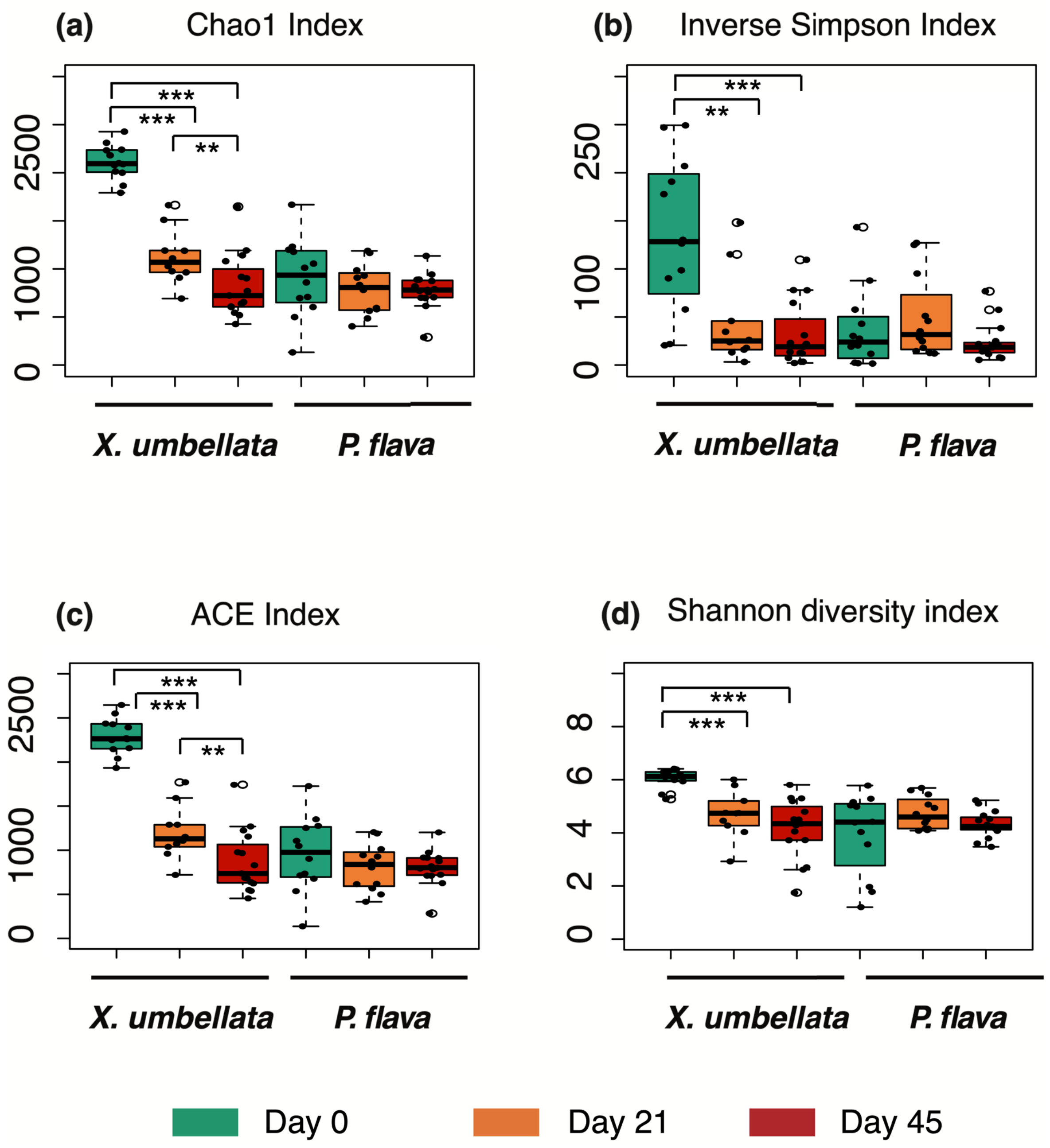

Fig. S2 Alpha diversity of bacterial communities in corals *X. umbellata* and *P. flava*. The asterisks indicate statistically significant differences compared to day 0 for each species separately (*$p < 0.05$; **$p < 0.01$; ***$p < 0.001$). (a) Chao1 index. (b) Inverse Simpson Index. (c) ACE index. (d) Shannon diversity index.

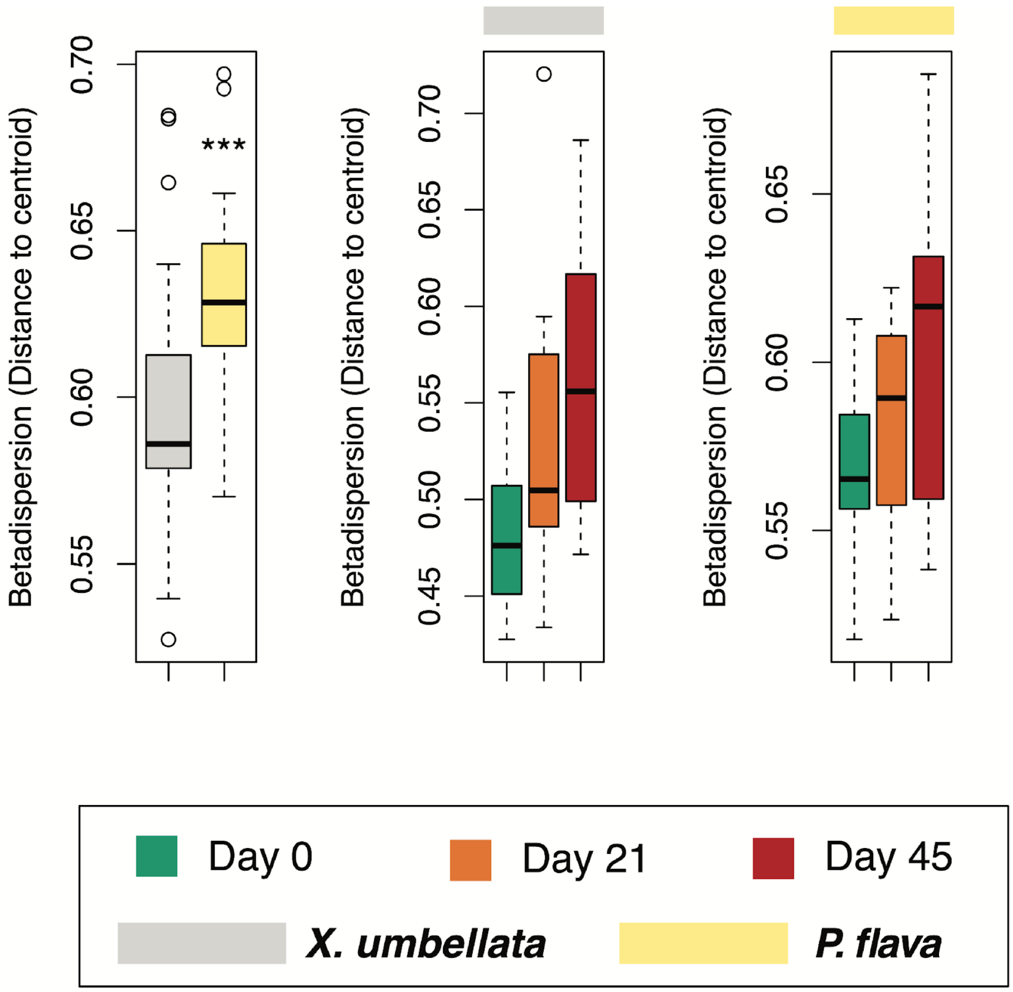

Fig. S3 Multivariate dispersion of bacterial communities in corals *X. umbellata* and *P. flava*. The asterisks indicate statistically significant differences between groups (*$p < 0.05$; **$p < 0.01$; ***$p < 0.001$).

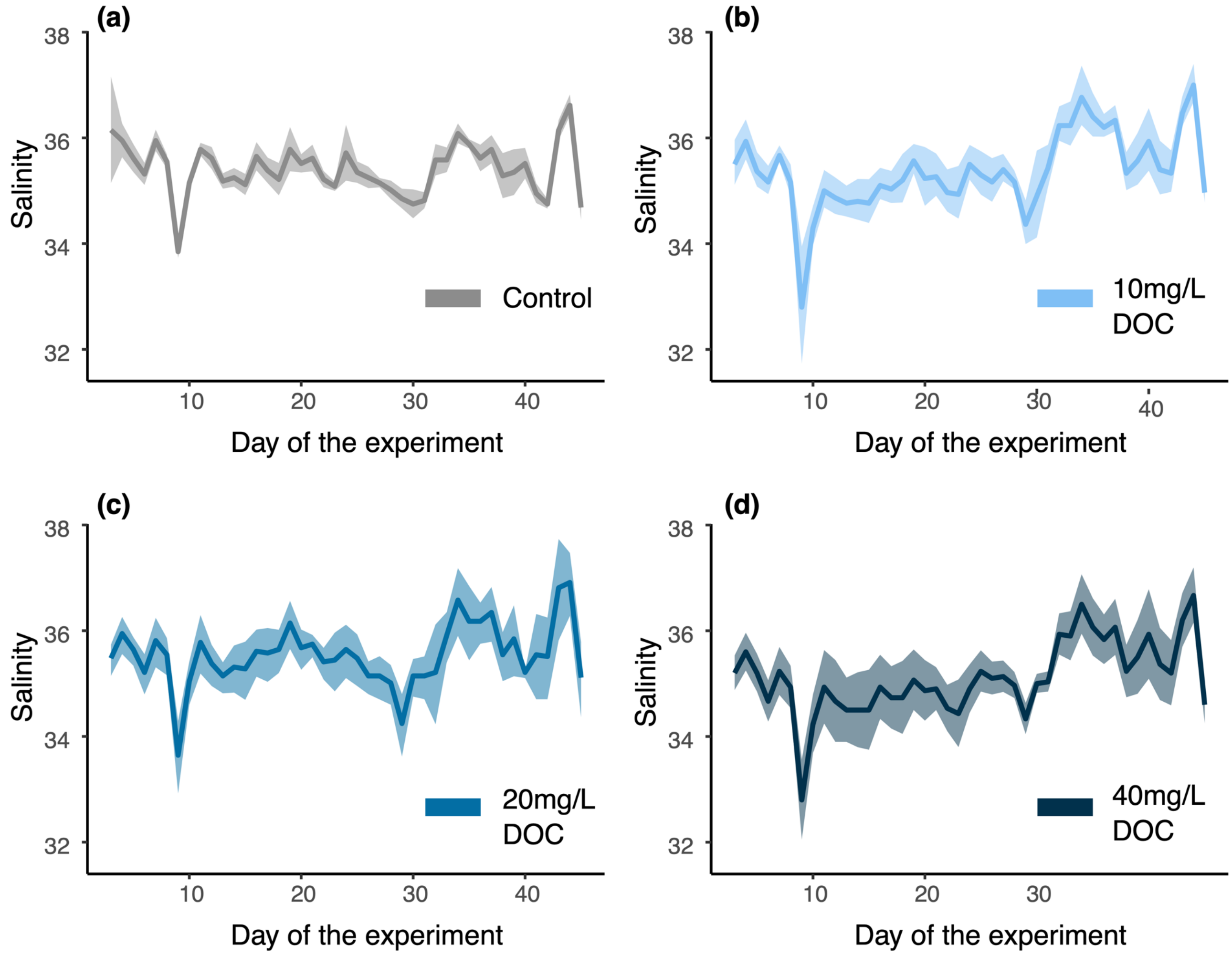

Fig. S4 The fluctuations in salinity (daily measurement) throughout the experiment. Values are shown by means ± SE for different treatments: (a) control; (b) low DOC (10 mg L^{-1}); (c) medium DOC (20 mg L^{-1}); (d) high DOC (40 mg L^{-1}).
